# A Case of Acquired Hemophilia A and Congenital Hemophilia B

**DOI:** 10.7759/cureus.30324

**Published:** 2022-10-15

**Authors:** Julia C Fortier, Shiyi S Pang, Sam Amofa-Ho, Neil S Harris, Marc Zumberg

**Affiliations:** 1 College of Medicine, University of Florida, Gainesville, USA; 2 Internal Medicine, University of Florida, Gainesville, USA; 3 Pathology, Immunology and Laboratory Medicine, University of Florida, Gainesville, USA; 4 Hematology and Oncology, University of Florida, Gainesville, USA

**Keywords:** factor replacement therapy, factor ix deficiency, acquired factor xiii deficiency, hemophilia b, acquired hemophilia a (aha)

## Abstract

Congenital hemophilia B is a rare, inherited X-linked bleeding disorder caused by a deficiency of factor IX (FIX). Acquired hemophilia A is a rare, acquired bleeding disorder which presents as new onset bleeding in older adults due to the development of autoantibodies against factor VIII (FVIII). This report describes the management of a patient with congenital hemophilia B and acquired hemophilia A. We highlight the limitations in maintaining FVIII levels using factor replacement alone and the need for escalating treatment such as rituximab and prednisone in patients with acquired hemophilia A. This case demonstrates the importance of continuing to pursue alternative diagnoses when existing ones do not explain the full clinical picture and laboratory data is inconclusive.

## Introduction

Acquired hemophilia A, a bleeding disorder due to the development of autoantibodies against factor VIII (FVIII), occurs in 1.48 per million persons per year and has an increasing incidence with age [1]. It typically occurs in older individuals without any personal or family history of bleeding. Subcutaneous bleeding is the main presenting symptom although soft tissue, muscle, and joint bleeds, similar to congenital hemophilia patients, can be seen [2]. The etiology of FVIII directed autoantibodies is often unknown, with 50% of the cases occurring without an obvious cause, and the remainder associated with autoimmune diseases, cancer, pregnancy, or idiosyncratic drug reactions [3]. Hemophilia B is an X-linked inherited factor IX (FIX) deficiency with an incidence of one in 30,000 male births [4]. The severity of the disease can be mild (6%-40% FIX activity), moderate (1%-5%), or severe (<1%). To our knowledge, we report the first case of a patient with congenital hemophilia B to develop acquired hemophilia A.

## Case presentation

A man in his mid-70s with known mild hemophilia B (baseline FIX 17%-25%) was not routinely followed by a hematologist. He endorsed several male family members who also carried a diagnosis of hemophilia B. Our patient’s only history of bleeding occurred after a tooth extraction three years prior. At that time, the patient had not informed his surgeon of his diagnosis of hemophilia B prior to the operation. Post-operatively, he presented to the emergency department with oral bleeding and a FIX activity level of 22%; he was treated with FIX concentrate. His medical history is otherwise significant for colon cancer treated with colectomy 17 years ago, though he could not recall if he received factor concentrate prior or if he had any bleeding related to this surgery. He had no evidence of cancer recurrence based on recent imaging. 

He was in his usual state of health when he presented to an outside hospital for evaluation of three months of groin pain in the absence of trauma. He was found to have an iliopsoas hematoma and his FIX activity was 28%. He improved with FIX replacement and was discharged home.

**Figure 1:**
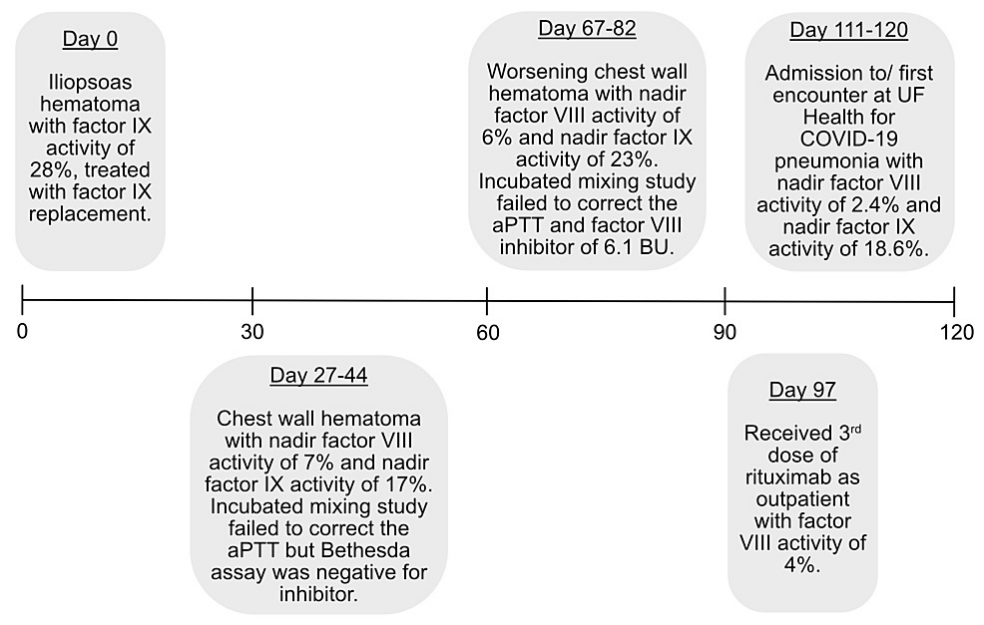
Timeline of various hospital admissions and outpatient encounters aPTT: activated partial thromboplastin time; BU: Bethesda units.

**Figure 2:**
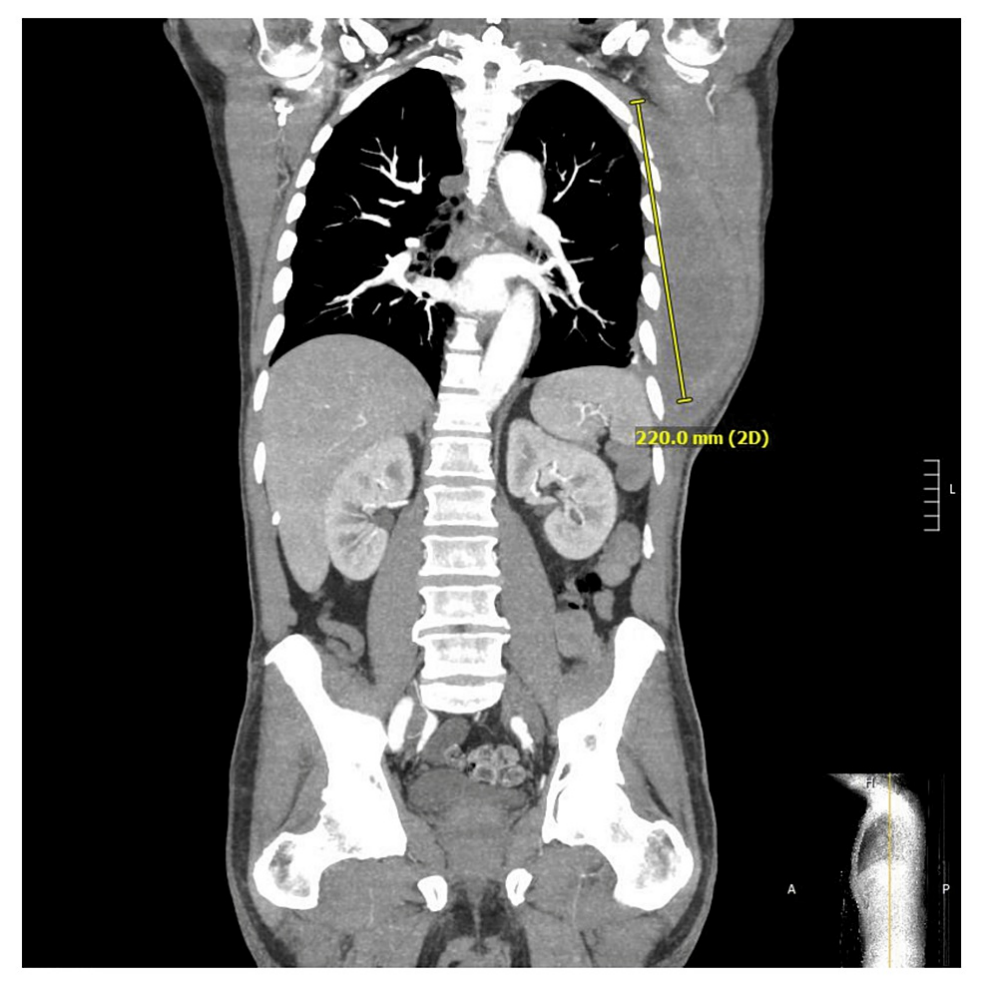
Left lateral chest wall hematoma Left lateral chest wall hematoma measuring approximately 13.4 x 6.7 x 22 cm and extending superiorly to the level of the scapular spine.

Less than one month later, he was admitted to the same outside hospital with a rib fracture and left-sided chest wall hematoma that occurred after a fall (Figures 1-2). His admission labs were significant for a hemoglobin of 8.7 g/dL (baseline unknown; ref range: 12.6-16.7), white blood cell count of 3.7 thou/cumm (ref range: 4.4-10.5), activated partial thromboplastin time (aPTT) of 61.7 s (ref range: 26.2-37.2), prothrombin time (PT) of 15.8 s (ref range: 9.5-13.3 s), FVIII activity 21% (ref range: 55-200%), and FIX activity 39% (ref range: 65-150%). He received two units of packed red blood cells. In addition, the patient was found to have elevated kappa (3.82 mg/dL; ref range: 0.33-1.94) and lambda (4.56 mg/dL; ref range: 0.57-2.63) serum free light chains. A bone marrow biopsy was unrevealing, however, he experienced persistent bleeding from his bone marrow biopsy site.

Incubated mixing studies failed to correct the aPTT but Bethesda assays did not reveal an inhibitor to FVIII or FIX. ANA titer was > 1:640 and speckled, but the patient lacked signs of rheumatologic disease, and autoimmune battery was otherwise negative. His FVIII activity remained around 24% and FIX activity around 20% during the two-and-a-half-week hospitalization. He was administered periodic FIX replacement with good response. He did experience one episode of recurrent bleeding from the bone marrow biopsy site when his FVIII activity dropped to 7%. This required high-dose FVIII replacement, aminocaproic acid (1,000 mg oral every 12 hours for three days), and suture placement with resolution of bleeding. In addition to bone marrow biopsy and rheumatologic workup, serum protein electrophoresis (SPEP), prostate-specific antigen (PSA), and computed tomography (CT) scan of the chest, abdomen, and pelvis were unrevealing. FVIII and FIX full gene sequencing did not reveal any known pathogenic mutations but did demonstrate a variant of unknown significance (VUS), c.1080C>G, p.Phe360Leu, in FIX. The patient was discharged home with conservative management for the rib fracture with recommended outpatient hematology follow up. He did not receive subsequent factor replacement.

Less than one month later, he was admitted to the same outside hospital for worsening left-sided chest wall hematoma. Mixing studies once again failed to correct the aPTT, but now a Bethesda assay revealed an FVIII inhibitor titer of 3.3 Bethesda units (BU). He was started on scheduled FVIII replacement, aminocaproic acid, and required multiple packed red blood cell transfusions. His admission labs were significant for a hemoglobin of 6.0 g/dL (baseline unknown; ref range: 12.6-16.7), white blood cell count of 13 thou/cumm (ref range: 4.4-10.5), aPTT of 42.5 s (ref range: 26.2-37.2), PT of 14.7 s (ref range: 9.5-13.3 s), FVIII activity 119% (ref range: 55-200%; drawn after FVIII replacement), and FIX activity 23% (ref range: 65-150%). During the admission, he received occasional FIX replacement with good response. FVIII activity decreased to a nadir of 6% despite escalating replacement doses. A repeat Bethesda study revealed an escalating FVIII inhibitor titer of 6.1 BU. He was started on FVIII inhibitor bypassing activity (FEIBA), 7,959 units IV twice daily for five days, and given two weekly doses of rituximab 750mg/m2. The bleeding stabilized and he was discharged with instructions to follow up with hematology.

Three weeks later, he received his 3rd dose of rituximab as an outpatient and his FVIII activity level was found to be 4% and FIX activity was 17%, without clinical signs of bleeding. Two weeks later and prior to his 4th scheduled dose of rituximab, he was admitted to UF Health with confusion and weakness, and found to have COVID-19 pneumonia. He was treated with two doses of remdesivir. On admission, his hemoglobin was 8.1 g/dL (ref range: 13-16.5), aPTT was 99 s (ref range: 25-38), FVIII level was 2.4% (ref range: > 54) and FIX 18.6% (ref range: 78-184), without active bleeding. FVIII inhibitor measured at 5.2 BU and an inhibitor to FIX was not detected. This was consistent with the previous findings of inherited hemophilia B with an acquired FVIII inhibitor. No FVIII or IX replacement or bypassing agents were required during this hospitalization as he did not have any bleeding. He was started on prednisone 1 mg/kg/d for treatment of the acquired FVIII inhibitor and prophylactic sulfamethoxazole-trimethoprim 800-160 mg oral three times weekly. Nine days later, aPTT was 62 s and FVIII activity increased to 51%. The patient was discharged on 80 mg prednisone PO daily with close follow-up with his local hematologist. Three weeks post discharge, his FVIII level was 212%.

## Discussion

To our knowledge, this is the first report of a patient developing acquired hemophilia A in the setting of known congenital hemophilia B. Coinheritance of hemophilia A and B has been reported in select patients and treated with replacement of both factors [5]. One confounder of our case was the initial incubated mixing study which supported an inhibitor, but a Bethesda assay did not detect an FVIII inhibitor at the outside hospital. Despite these conflicting results, his subsequent positive Bethesda assays and new onset decreased FVIII activity, despite repeated doses of FVIII, favored the diagnosis of an acquired hemophilia A. This highlights the limitations of certain laboratory assays. For example, inhibitors against FVIII are time and temperature dependent, and immediate mixing tests may show a false correction if time is not allowed for inhibitors to bind [6].

The driver of his FVIII inhibitor remains unknown, as the workup for malignancy and rheumatologic disease was negative. Acquired hemophilia A is idiopathic in half of the cases, but can be associated with malignancy, autoimmune disorders such as rheumatoid arthritis, infections, and certain medications [7]. In patients with inhibitor titers greater than 5 BU, FVIII replacement alone may not be sufficient, which explains our patient’s lack of response to FVIII replacement and the need for additional bypassing therapies. In patients with acquired hemophilia, prothrombin complex concentrates (PCCs), recombinant factor VIIa, and porcine factor VIII (pFVIII) are often used to secure hemostasis [7]. Rituximab alone has been associated with a longer time to FVIII recovery compared to that of corticosteroids alone. The combination of corticosteroids and cyclophosphamide may lead to quicker eradication of an FVIII inhibitor but is associated with a higher risk of infection [7,8]. Our patient’s FVIII levels appeared to respond rapidly to prednisone; however, his rising factor levels could also have been secondary to the recent rituximab administration, which can take weeks to become effective [9].

Next-generation sequencing of the F9 gene revealed no known pathogenic mutations but did reveal a VUS. This must be interpreted within the assay’s limitations. Though sequencing identifies a causative mutation in roughly 97% of patients with hemophilia B, the remaining patients lack such a mutation as certain variants are difficult to detect with current sequencing technologies, or have not yet been identified in the pathogenesis of hemophilia [10]. The exact nucleotide substitution causing ann F9 VUS in our patient that resulted in a phenylalanine to leucine substitution at position 360 had not yet been reported, but is similar to existing substitutions associated with hemophilia B. A different nucleotide substitution resulting in the same Phe360Leu amino acid change has been reported to be associated with hemophilia B [11]. This phenylalanine is located on a bend in the secondary structure within the serine protease domain of FIX [12]. Due to different numbering of the residues (Human Genome Variation Society vs legacy numbering), the same mutation is referred to as Phe314Leu in a separate report and was associated with a mild bleeding phenotype, similar to that of our patient. Other nucleotide substitutions at the same residue have also been reported resulting in the following amino acid substitutions: Phe360Ile, Phe360Cys, and Phe360Ser [13-14]. Taken together, these reports suggest that substitutions of amino acid residue Phe360 are not well-tolerated, and the variant at this locus was likely the case of our patient’s congenital hemophilia B.

## Conclusions

This report describes a patient with congenital hemophilia B and acquired hemophilia A, and highlights the challenges in diagnosing and treating the latter. In instances where FVIII replacement alone is not sufficient to maintain factor levels, escalating treatment such as rituximab and prednisone should be considered. In the right clinical circumstances, such as an older adult with new-onset, unexplained bleeding, acquired hemophilia should be considered, even in patients with a known, inherited bleeding disorder. Given the differences in pathogenesis, there is no correlation between the presence of congenital hemophilia B and acquired hemophilia A, but the two can occur concurrently by chance. Though we report the first case of concomitant acquired hemophilia A and congenital hemophilia B, reports of combined inherited hemophilia and Von Willebrand’s disease have been documented. The key to making this diagnosis, as well as others where the existing diagnosis does not explain the complete clinical scenario, was for clinicians to cede any anchoring bias in order to consider additional causes. In addition, this case reinforces the importance of careful review of outside laboratory data and genetic analysis, or sequencing reports as in our case.
